# Hypolipidemic and Hepatoprotective Effects of 5-(4-(3-thioxo-3H-1,2-dithiol-5-yl)phenoxy)Pentanoic Acid (TDPPA) on Hyperlipidemic Mice

**DOI:** 10.2174/0115734064425298250923050512

**Published:** 2025-10-08

**Authors:** Ting Li, Manjun Liu, Qiuzhen Yuan, Wenjing Shen, Xueyou Chen, Lei Zhang, Yundong Xie

**Affiliations:** 1 Department of Pharmaceutical, Shaanxi Institution of Traditional Chinese Medicine, Shaanxi Traditional Chinese Medicine Hospital, Xi'an, China;; 2 Department of Pharmacy, Pengzhou City Fourth People's Hospital, Pengzhou Mental Health Center, Pengzhou, China;; 3 Department of Pharmacy, Zigong Hospital of Traditional Chinese Medicine, Zigong, China;; 4 School of Pharmacy, Shaanxi University of Chinese Medicine, Xianyang, China

**Keywords:** 5-(4-(3-thioxo-3H-1, 2-dithiol-5-yl)phenoxy)pentanoic acid, anti-inflammation, antioxidation, hepatoprotection, hypolipidemia, cardiovascular diseases

## Abstract

**Introduction:**

Hyperlipidemia is a prevalent condition that accelerates the development of cardiovascular diseases. Traditional treatments targeting lipid regulation often have limitations, such as hepatotoxicity. This study investigates the dual action of a novel compound, 5-(4-(3-thioxo-3H-1,2-dithiol-5-yl)phenoxy)pentanoic acid (TDPPA), in reducing lipid levels and protecting the liver.

**Methods:**

TDPPA was synthesized and structurally confirmed by ^1^H-NMR, ^13^C-NMR, and HR-MS. Its lipid-lowering efficacy was first assessed in Triton WR-1339-induced acute hyperlipidemic mice. Mechanistic studies were then conducted in a high-fat emulsion-induced chronic hyperlipidemia model, incorporating histopathological analysis of the liver (H&E and Oil Red O staining). Liver index, serum lipid panels, hepatic function markers, H_2_S content, oxidative stress parameters, and pro-inflammatory cytokines were quantified* via *ELISA, while the interaction between TDPPA and PPAR-α was evaluated by molecular docking and Western blotting.

**Results:**

TDPPA significantly reduced serum triglyceride (TG), total cholesterol (TC), and low-density lipoprotein cholesterol (LDL-C) in both acute and chronic models, while increasing high-density lipoprotein cholesterol (HDL-C). Histology revealed marked reductions in hepatic lipid accumulation and inflammatory infiltration. Biochemical assays showed decreases in AST and ALT, enhanced antioxidant capacity (higher SOD and H_2_S, lower MDA), and suppression of TNF-α, IL-6, and IL-1β. Molecular docking and Western blot analysis indicated that these effects were associated with upregulation of PPAR-α protein expression.

**Discussion and Conclusion:**

TDPPA demonstrates potent lipid-lowering, antioxidant, and anti-inflammatory activities, likely through a dual mechanism involving PPAR-α activation and H_2_S-mediated hepatoprotection. These findings position TDPPA as a promising therapeutic candidate for hyperlipidemia with the benefit of liver protection.

## 1 INTRODUCTION

Hyperlipidemia (HLP) represents a complex metabolic disorder characterized by dysregulated lipid profiles, including elevated serum concentrations of total cholesterol (TC), triglycerides (TG), low-density lipoprotein cholesterol (LDL-C), and reduced high-density lipoprotein cholesterol (HDL-C) levels. Clinically, HLP manifests in four distinct phenotypes, including hypercholesterolemia, hypertriglyceridemia (HTG), high LDL-C, and mixed-type hyperlipidemia [1]. The development of HLP involves multiple aspects of lipid metabolism, primarily including enhanced intestinal absorption of dietary cholesterol, enhanced endogenous lipid synthesis, and impaired lipid transport and catabolic pathways. The liver, as the main site of both lipid synthesis and catabolism, plays a major role in the pathogenesis of HLP. When hepatic lipid metabolism becomes overwhelmed by excessive circulating lipids, lipids accumulate in the liver, leading to hepatic injury and resulting in non-alcoholic fatty liver disease (NAFLD), including non-alcoholic fatty liver, non-alcoholic steatohepatitis, and NAFLD-related cirrhosis [2]. Conversely, NAFLD-induced hepatocyte damage further exacerbates HLP [3]. Beyond liver injury, HLP plays a key role in the progression of cardiovascular diseases, serving as an independent risk factor for cerebrovascular diseases and atherosclerosis [4].

Additionally, HLP is considered the link to neurodegenerative diseases, including Alzheimer's disease and Parkinson's disease [5]. With the improvement of people’s living standards and changes in dietary habits, the prevalence of HTG is increasing, particularly in Asian countries, where HTG rates are significantly higher than hypercholesterolemia [6]. Reports have demonstrated that increased TG levels are strongly associated with a high risk of atherosclerotic cardiovascular disease (ASCVD), and severe elevation of TG levels may further heighten the risk of TG-induced pancreatitis [7]. Therefore, effective prevention and treatment of HTG remain an urgent medical priority.

Peroxisome proliferator-activated receptor alpha (PPAR-α) agonists, such as fibrates, serve as first-line therapeutic agents for HTG [8]. Although PPAR-α agonists demonstrate significant clinical efficacy, substantial adverse reactions, particularly hepatotoxicity, may occur after long-term administration [9]. In clinical practice, liver function parameters in patients must be closely monitored after 4 to 8 weeks of fibrates administration, with particular attention to the potential for hepatic injury [10]. Research indicates that the adverse effects of fibrates are mechanistically linked to oxidative stress induced by PPAR-α pathway activation [11]. Consequently, novel pharmacological agents capable of simultaneously reducing TG levels while suppressing oxidative stress to mitigate hepatic injury hold significant clinical relevance.

Hydrogen sulfide (H_2_S), a gaseous signaling molecule that can freely diffuse across the cell membranes, exerts diverse biological effects through activation of multiple cellular targets [12]. Studies demonstrate that H_2_S participates in various pathophysiological processes, including anti-inflammation, antioxidant activity, vasodilation, cardiac protection, and neuroprotection [13]. It has been reported that there is a positive correlation between H_2_S levels and HDL-C levels in adults, while a negative correlation exists with the LDL-C levels [14]. Recent studies have further demonstrated that elevated levels of H_2_S are associated with a reduction in blood lipid levels [15, 16]. Developing novel therapeutics with combined lipid-lowering, antioxidant, and anti-inflammatory properties holds promise for preventing HLP-induced hepatic injury. Such novel H_2_S-releasing drugs, capable of exerting multiple protective effects, may provide new therapeutic options for HLP treatment.

In our previous studies, as shown in Fig. (**1**), compounds 6 and T5, which possess H_2_S-releasing properties, were found to exhibit significant TG-lowering, anti-inflammation, and antioxidant effects, demonstrating significant liver-protective activity in the context of HLP [17, 18]. Incubation experiments using hepatic mitochondria revealed that these two compounds were metabolized to 5-(4-(3-thioxo-3H-1,2-dithiol-5-yl)phenoxy)pentanoic acid (TDPPA) (Fig. **1**). Therefore, this study aimed to evaluate the potential effects of TDPPA on attenuating TG accumulation and conferring hepatic protection.

The synthesis of TDPPA was initially conducted, and subsequently, the Triton WR-1339-induced acute HLP mouse models were employed to evaluate its dose-dependent lipid-lowering activity. Furthermore, the mechanisms of TDPPA were assessed in a high-fat emulsion-induced chronic hyperlipidemic mouse model, and its impact on the liver was systematically evaluated, including liver index, histopathology, liver function markers, H_2_S content, oxidative stress indicators, and pro-inflammatory cytokines. This study aims to provide a potential candidate compound with both TG-lowering and hepatoprotective functions.

## 2 MATERIALS AND METHODS

### 2.1 Materials and Instruments

All chemical reagents and experimental materials were sourced from certified commercial suppliers with specified purity grades: 3,4-methylenedioxyphenyl-2-propanone (purity: 99%) was purchased from Hubei Saint-Bio Technology Co., Ltd, 5-bromovaleric acid ethyl ester (purity: 98%) from Sann Chemical Technology (Shanghai) Co., Ltd, Pyridine hydrochloride (purity: 98%) from Aladdin Reagent Co., Ltd. Column chromatography silica gel and thin-layer plates were supplied by Qingdao Marine Chemical Reagent Factory, while organic solvents for the synthesis were procured from Tianjin Damao Chemical Reagent Factory. Biochemical assay kits from Nanjing Jiancheng Bioengineering Institute (China) were used for biomarker measurements in serum and liver tissues, including ALT (Cat# C010-2-1), AST (Cat# C009-2-1), SOD (Cat# A001-3-1), MDA (Cat# A003-1-1), TNF-α (Cat# H052-1), and IL-6 (Cat# H007-1-2). For Western blotting, anti-PPARα antibody (Cat# ab314112; Abcam, UK) and HRP-conjugated goat anti-rabbit IgG (H+L) secondary antibody (Cat# SA00001-2; ProteinTech, USA) were employed. Triton WR-1339 was obtained from Sigma-Aldrich (USA).

Instrumentation was carried out using the following instruments: nuclear magnetic resonance hydrogen and carbon spectra were acquired on a nuclear magnetic resonance spectrometer (model: Bruker Avance NEO 400MHz), while high-resolution mass spectrometry was performed by a liquid chromatography-mass spectrometry (model: Thermo Scientific Q Exactive Orbitrap), and melting points were measured by a melting point apparatus (model: Shanghai X-4A).

### 2.2 Synthesis and Structure Characterization of TDPPA

The synthesis of 5-(4-hydroxyphenyl)-3H-1,2-dithiole-3-thione (ATT-OH) and ethyl 5-(4-(3-thioxo-3H-1,2-dithiol-5-yl)phenoxy)pentanoate was conducted according to the previous protocols [17]. Subsequently, 0.50 g (1.41 mmol) ethyl 5-(4-(3-thioxo-3H-1,2-dithiol-5-yl)phenoxy)pentanoate was dissolved in 15 mL of 1,4-dioxane in a flask, and 5 mL of 37% hydrochloric acid was added. The mixture was refluxed at 60°C, and its progression was monitored by thin-layer chromatography (TLC). Upon completion, the mixture was quenched with deionized water (40 mL), resulting in precipitation of the solid. The product was extracted with 20 mL of ethyl acetate, and impurities were removed by adjusting the pH to 7.0. After solvent evaporation under reduced pressure, TDPPA was obtained by recrystallization from absolute ethanol as a red solid (0.28 g, 61.0% yield), with a melting point of 132.3-132.6°C. HRMS (ESI, negative mode): m/z calculated for C_14_H_14_O_4_S_4_ [*M*-H]-: 325.0027, found 325.0043. ^1^H-NMR (400 MHz, DMSO-*d*_6_) *δ*: 12.04 (s, 1H, -COOH), 7.88-7.86 (m, 2H, Ar-H), 7.77 (s, 1H, -CH=), 7.08-7.06 (m, 2H, Ar-H), 4.09-4.06 (t, *J* = 6.2 Hz, 2H, -OCH_2_-), 2.29-2.27 (t, *J* = 7.3 Hz, 2H, -CH_2_CO-), 1.77-1.63 (m, 4H, -CH_2_CH_2_-). ^13^C-NMR (100 MHz, DMSO-*d*_6_) *δ*: 215.21, 174.33, 173.23, 162.55, 134.56, 129.48, 124.02, 115.95, 68.12, 33.56, 28.29, 21.56.

### 2.3 Animal Experiments

Male Kunming mice (20 ± 2 g, 4 weeks old) were obtained from Chengdu Dashuo Experimental Animal Co., Ltd (Licence No. SCXK (Sichuan) 2015-030). All mice were acclimatized for 3 days under standard laboratory conditions (22 ± 1°C, 50 ± 5% humidity), with free access to food and water.

For experiment 1, the mice were randomly divided into the following groups (n = 8): control (0.5% CMC-Na solution), model (0.5% CMC-Na solution), Clofibrate (65 mg/kg in 0.5% CMC-Na), and TDPPA groups (7.5, 15, 30 mg/kg in 0.5% CMC-Na). On day 6 of oral gavage administration, acute hyperlipidemic mouse models were induced in all groups by intravenous injection of Triton WR-1339 (400 mg/kg in normal saline, 80 mg/mL) except for the control. Blood samples were collected from the mice 24 h after model induction.

For experiment 2, chronic HLP in mice was induced by administration of high-fat emulsion diets, consisting of the following components per 100 mL: Cholesterol (10.0 g), lard (20.0 g), sodium cholate (2.0 g), tween-80 (20.0 mL), propylene glycol (30.0 mL), methylthiouracil (1.0 g), and purified water to the volume [19]. The mice were randomly divided into four groups (n = 8): control (distilled water), model (high-fat emulsion, 25 mL/kg/day), Clofibrate (high-fat emulsion (25 mL/kg/day) + Clofibrate (10 mg/mL in 0.5% CMC-Na)), and TDPPA (high-fat emulsion (25 mL/kg/day) + TDPPA (15 mg/mL in 0.5% CMC-Na)). All animals were treated by oral gavage daily for 28 consecutive days under standardized feeding conditions.

Ethical approval for this study was obtained from the Institutional Animal Ethics Committee of Shaanxi Institution of Traditional Chinese Medicine (Ethics Review Approval No. 85 (2023)) prior to initiation of the study. All animal experimental procedures were performed in accordance with the US National Research Council's “*Guide for the Care and Use of Laboratory Animals*”. This study adhered to internationally accepted standards for animal research, following the 3Rs principle. The ARRIVE guidelines were employed for reporting experiments involving live animals, promoting ethical research practices.

### 2.4 Biochemical Measurements

About 12 hours after treatment, blood samples were obtained *via* orbital sinus puncture. Subsequently, the serum was isolated by centrifuging at 3500 g for 10 minutes at 4°C. Lipid profiles (TC, TG, LDL-C, HDL-C), hepatic function markers (AST, ALT), oxidative stress parameters (SOD, MDA, H_2_S), and serum inflammatory cytokines (TNF-α, IL-1β, and IL-6) were quantified using commercial assay kits following the manufacturer’s protocols.

### 2.5 Histopathological Observation

The liver tissues were dissected after cervical dislocation, rinsed with physiological saline, dried with blotting paper, weighed, and fixed in 4% paraformaldehyde for histological analysis. Oil Red O and H&E staining were performed separately to observe the liver's histomorphology, according to established protocols [20]. Stained sections were examined under electron microscopy for photographic documentation. For histological scoring, five non-overlapping fields from each section were randomly selected, and the mean value was used for analysis. Inflammatory changes were assessed according to the criteria outlined by Shen *et al*. [21] as follows: score 0: no inflammatory foci or necrosis; score 1: less than 1/4 of the field affected; score 2: 1/4-1/2; score 3: 1/2-3/4; and score 4: greater than 3/4. Oil Red O-positive areas were quantified using ImageJ (NIH, USA). For this, images from (another set of) five randomly selected fields were converted into RGB stacks, and the red channel was isolated to highlight lipid staining. A uniform threshold was applied to segment positive regions, and the stained area was calculated as a percentage of the total tissue area in each field. The mean percentage per sample was used for statistical analysis.

### 2.6 Molecular Docking

Molecular docking was conducted individually for TDPPA and Clofibrate against the PPAR-α receptor. The docking results were visualized using PyMOL software (https://www.pymol.org/). The molecular structures of TDPPA and Clofibrate were obtained from PubChem (https://pubchem.ncbi.nlm.nih.gov/) and energy-minimized using a PyMOL plugin. The crystal structure of PPAR-α was downloaded from the RCSB Protein Data Bank (PDB ID: 6KAX, https://www.rcsb.org/). Before docking, the target protein structure was preprocessed using AutoTools to remove water molecules and add hydrogen atoms. The GetBox plugin in PyMOL was used to predict the receptor’s active binding pockets. Docking simulations were carried out using AutoDock Vina. For each ligand, the docking pose with the lowest binding energy was selected. The optimal docking poses were visualized in PyMOL for subsequent interaction analysis.

### 2.7 Western Blot Analysis

The expression of PPAR-α in liver tissues was assessed by Western blot analysis. Tissues were collected and homogenized in pre-chilled RIPA buffer (Beyotime Biotech, Jiangsu, China), supplemented with protease/phosphatase inhibitors and PMSF. After incubation on ice for 20 minutes, the homogenates were centrifuged at 12,000 rpm for 15 minutes at 4°C to obtain the supernatant. Protein samples were denatured in SDS-PAGE loading buffer through boiling at 95°C for 5 min. Protein samples were separated by electrophoresis on 15% SDS-polyacrylamide gels and subsequently transferred onto nitrocellulose membranes with a wet transfer system. Membranes were blocked with 5% (w/v) non-fat dry milk in TBST for 1 h at room temperature, followed by incubation with primary antibodies (1:1000) diluted in blocking buffer overnight at 4°C. After three TBST washes, the membranes were incubated with HRP-conjugated secondary antibodies (1:2000) for 2 h at room temperature. Protein bands were visualized using ECL substrate and detected by an LAS-3000 imaging system (Fujifilm, Tokyo, Japan). Band intensity was quantified using Gel-Pro Analyzer software. All Western blot data presented in this study were derived from independent biological replicates to ensure statistical reliability.

### 2.8 Statistical Analysis

Statistical analyses were performed with GraphPad Prism software (v8.3.0), employing one-way ANOVA followed by Dunnett’s post-hoc test. Data were presented as mean ± SD, with statistical significance set at *P* < 0.05.

## 3 RESULTS

### 3.1 Chemistry

As illustrated in Scheme **1**, ATT was first mixed with pyridine hydrochloride and heated at 220°C for 30 minutes under reflux. After cooling and acidification, the crude product was purified by column chromatography (dichloromethane/methanol = 70:1) to yield ATT-OH. ATT-OH was subsequently dissolved in DMF and reacted with ethyl 5-bromopentanoate in the presence of K_2_CO_3_ and KI, giving ethyl 5-(4-(3-thioxo-3H-1,2-dithiol-5-yl)phenoxy)pentanoate. The ester intermediate was then dissolved in 1,4-dioxane and subjected to acid-catalyzed hydrolysis to yield the target compound, TDPPA. The structure and purity of TDPPA were confirmed by comprehensive spectroscopic analyses, including ¹H-NMR, ¹³C-NMR, and HRMS (as shown in the Supplementary material).

### 3.2 Effects of TDPPA on Serum Lipid Levels in Acute Hyperlipidemic Mice

As shown in Fig. (**2**), the model group exhibited significantly elevated serum levels of TG and TC (*P* < 0.01) compared with the control group, confirming the successful establishment of an acute hyperlipidemic model induced by Triton WR-1339. In comparison with the model group, low-, medium-, and high-doses of TDPPA treatments reduced TG levels by 8.3%, 36.5%, and 52.0% respectively, with the reductions at medium and high doses reaching significant differences (*P* < 0.05, Fig. **2A**). Notably, the TG-lowering effect of high-dose TDPPA exceeded that of the positive control drug, Clofibrate (-23.8%, Fig. **2A**). TDPPA also reduced TC levels by 11.8%, 36.6%, and 45.4% at low, medium, and high doses respectively (*P* < 0.05, Fig. **2B**), whereas Clofibrate (65 mg/kg) produced only a modest TC-lowering effect (5.4%). Collectively, these findings indicate that TDPPA exerts dose-dependent lipid-lowering effects on both TG and TC levels, with superior efficacy compared to Clofibrate in reducing TC.

### 3.3 Effects of TDPPA on Serum Lipid Levels in Chronic Hyperlipidemic Mice

In this study, a chronic HLP mouse model was established *via* intragastric administration of a high-fat emulsion. Compared with the control group, the model group exhibited significantly elevated serum TG, TC, and LDL-C levels, accompanied by a marked decrease in HDL-C, as shown in (Figs. **3A**-**D**). Four weeks of TDPPA administration (15 mg/kg) reduced TG, TC, and LDL-C levels by 68%, 46%, and 63% respectively, in model mice. Notably, HDL-C levels were significantly increased by 109% (*P* < 0.01, Fig. **3C**). In contrast, treatment with Clofibrate (65 mg/kg) resulted in a reduction of TG and LDL-C levels by 30% and 43%, respectively, while producing a 67% increase in HDL-C levels (*P* < 0.01, Fig. **3C**).

### 3.4 Effects of TDPPA on Atherosclerosis Index (AI) and Liver Index (LI) in Chronic Hyperlipidemic Mice

The Atherosclerosis Index (AI), calculated as the ratio of LDL-C to HDL-C, was significantly elevated in the model group compared with the control group (*P* < 0.01, Fig. **4A**). Notably, four weeks of oral TDPPA administration (15 mg/kg) resulted in an 85% reduction in the AI, indicating its therapeutic potential in lowering HLP-associated cardiovascular risk. As shown in Fig. (**4B**), body weight did not differ significantly among the groups at the end of the experiment (*P* > 0.05). Liver weight in the model group was higher than that in the control group (*P* < 0.01). Conversely, TDPPA (15 mg/kg) and Clofibrate (65 mg/kg) treatments markedly reduced liver weight (*P* < 0.01, Fig. **4C**). The liver index (LI), defined as the ratio of liver weight to total body weight, was also significantly elevated in the model group compared with the control (*P* < 0.01, Fig. **4D**). TDPPA administration led to a 16% reduction in LI (*P* < 0.01), whereas Clofibrate treatment produced a 26% reduction (*P* < 0.01).

### 3.5 Effects of TDPPA on Lipid Accumulation and Hepatic Structure in Chronic Hyperlipidemic Mice

Model mice developed marked hepatic steatosis, as shown in (Fig. **5A**), characterized by distinct foci of fat accumulation throughout the liver parenchyma. After four weeks of TDPPA (15 mg/kg) administration, lipid deposition was markedly attenuated, with fewer and smaller lipid-rich foci compared to the model group. H&E staining (Fig. **5B**) revealed that, in the control group, hepatic lobules maintained intact architecture, with uniform hepatocyte morphology and normal nuclei. In contrast, the model group exhibited hepatocellular ballooning, macrovesicular steatosis, and prominent inflammatory infiltration, consistent with hyperlipidemia-induced liver injury. TDPPA treatment significantly preserved hepatic architecture, restored hepatocyte arrangement, and reduced inflammatory infiltration, as reflected by a lower inflammation score (2.7 ± 0.4 in the model group *vs.* 1.1 ± 0.3 in the TDPPA group, *P* < 0.05, Fig. **5D**). Clofibrate (65 mg/kg) also improved hepatic histology, although the inflammation scores (1.4 ± 0.4) were numerically higher than those in the TDPPA group (1.1 ± 0.3), a difference that was not statistically significant (Fig. **5D**). Oil Red O staining (Fig. **5C**) confirmed severe steatosis in model hepatocytes, with a high density of red-stained lipid droplets (lipid area: 26% ± 5%, Fig. **5E**). TDPPA significantly reduced lipid accumulation (lipid area: 9% ± 1.1%, *P* < 0.05 *vs*. model), decreasing both droplet size and number (Fig. **5E**). Clofibrate produced a comparable reduction (11% ± 0.5%), although the lipid area remained slightly higher than that observed with TDPPA (Fig. **5E**). These results indicate that both agents alleviate hyperlipidemia-associated hepatic injury, with TDPPA showing a trend toward greater attenuation of lipid deposition and inflammatory infiltration.

### 3.6 Effects of TDPPA on Biochemical Parameters in Chronic Hyperlipidemic Mice

To investigate the potential mechanism underlying the hepatoprotective activity of TDPPA, we examined its effects on hepatic injury markers (AST and ALT), antioxidant indexes (SOD, MDA, and H_2_S), and pro-inflammatory cytokines (TNF-α, IL-1β, and IL-6) in mice with chronic HLP. As presented in (Fig. **6A**), the model group exhibited a notable elevation in AST and ALT levels compared with the control (*P* < 0.01), indicating substantial hepatic damage induced by high-fat emulsion administration. Four weeks of TDPPA (15 mg/kg) treatment resulted in a 62% reduction in AST levels (*P* < 0.01) and a 25% reduction in ALT concentrations (*P* < 0.05). Similarly, the model mice showed a significant decrease in SOD activity and an increase in MDA concentration compared with the control group (Fig. **6B**, *P* < 0.01), indicating a strong relationship between HLP and oxidative stress. TDPPA administration markedly elevated the serum SOD levels by 16% and reduced MDA content by 41% (*P* < 0.01). TDPPA also significantly increased H_2_S levels by 98% compared with the model group (*P* < 0.01), whereas Clofibrate (65 mg/kg) produced only a 12% increase, which was not statistically significant. As shown in Fig. (**6C**), concentrations of TNF-α, IL-1β, and IL-6 were markedly elevated in the model group (*P* < 0.01). TDPPA notably suppressed the inflammatory cytokine levels, decreasing TNF-α content by 28%, IL-1β levels by 39%, and IL-6 levels by 36% (*P* < 0.05). These biochemical findings strongly support the therapeutic potential of TDPPA for HLP-associated hepatic dysfunction, likely through its anti-inflammatory and antioxidant effects.

### 3.7 Effect of TDPPA on PPAR-α in Chronic Hyperlipidemic Mice

Molecular docking was performed using AutoDock Vina for TDPPA and Clofibrate with the PPAR-α receptor, and the results are presented in Fig. (**7**). Clofibrate (Fig. **7A**), which was used as a positive control, bound to multiple sites, with Binding Site 2 yielding an affinity of −6.4 kcal/mol, consistent with previous structural analyses (Kamata *et al*., 2020). For TDPPA (Fig. **7B**), multiple binding pockets were identified, with Binding Site 2 exhibiting the most favorable binding affinity (−6.9 kcal/mol). The primary interactions were mediated by Tyr334, Cys275, and Leu258 through a combination of hydrophobic contacts and hydrogen bonding (Fig. **7B**), suggesting stable and specific recognition of the ligand by the receptor. Collectively, these results indicate that TDPPA possesses a robust binding capacity toward PPAR-α, comparable to that of the established positive control, supporting its potential as a promising modulator of PPAR-α activity for applications in lipid metabolism.

Given the strong predicted binding of TDPPA to PPAR-α, experimental validation was undertaken to determine whether such interactions translate into modulation of receptor expression *in vivo*. Western blot analysis was employed to quantify PPAR-α protein levels in liver tissues from HLP mice after treatment (Fig. **8**). In the disease model group, PPAR-α protein abundance was markedly reduced compared with the control, paralleling increases in serum TG and TC. Administration of TDPPA (15 mg/kg) significantly elevated PPAR-α expression (*P* < 0.05) relative to the model group. Similarly, Clofibrate treatment (65 mg/kg) led to a significant upregulation of PPAR-α protein (*P* < 0.05). These findings provide both computational and experimental evidence that TDPPA binds to PPAR-α and enhances its expression, supporting its potential role in regulating lipid metabolism through PPAR-α-mediated pathways.

## 4 DISCUSSION

The present study demonstrates that the chemically synthesized metabolite TDPPA exhibits multifunctional therapeutic properties, including lipid-lowering effects, H_2_S-releasing capacity, and both antioxidative and anti-inflammatory activities, collectively contributing to its hepatoprotective efficacy under hyperlipidemic conditions. These effects were observed without signs of hepatotoxicity during the treatment period, underscoring its therapeutic potential. To investigate the anti-hyperlipidemic mechanism of TDPPA, we first employed the Triton WR-1339-induced acute hyperlipidemia model because it is straightforward to perform, produces a rapid and reproducible elevation in serum triglyceride and cholesterol levels, and is widely used for the rapid screening of lipid-modulating agents [22]. To further explore the chronic effects, a murine model of chronic HLP induced by high-fat emulsion feeding was applied. This particular model was selected due to its close resemblance to human dietary patterns and the absence of “anorexia”, which can be a concern in prolonged high-fat feeding protocols [23].

Chronic HLP induces hepatic lipid accumulation and alters redox homeostasis, thereby amplifying the production of reactive oxygen species (ROS), which in turn initiates oxidative stress reactions [24, 25]. This oxidative stress, driven by inflammatory responses, not only impairs the normal oxidative metabolism of lipids but also induces membrane phospholipid peroxidation, resulting in hepatocyte injury [20], thereby forming a self-perpetuating cycle [26]. Research indicates that chemicals possessing antioxidant and anti-inflammatory properties can confer significant hepatoprotection by regulating ROS and inflammatory cytokines [27]. Our findings reveal that TDPPA is capable of mediating H_2_S liberation in hyperlipidemic mice, effectively restoring H_2_S concentrations during dyslipidemia. The release of H_2_S may reduce peroxidation levels under hyperlipidemic conditions by directly scavenging oxidative molecules generated by intracellular reactive oxygen and nitrogen species, including H_2_O_2_ and superoxide anions [28]. As we observed in the present study, TDPPA exhibited pronounced antioxidant abilities, as evidenced by elevated SOD levels and reduced MDA levels, alongside anti-inflammatory effects reflected by decreased pro-inflammatory cytokines (TNF-α, IL-1β, and IL-6). Both the antioxidant and anti-inflammatory responses observed in this study have been known to be modulated by H_2_S in previously established experimental models [29, 30]. Therefore, we hypothesize that TDPPA, by releasing H_2_S, may exert hepatoprotective effects, while promoting lipid metabolism and reducing blood lipids, thereby ameliorating oxidative stress damages and inflammatory responses under HLP conditions.

Previous research has confirmed that PPAR-α plays a crucial regulatory role in the expression of key genes involved in lipoprotein metabolism, ultimately leading to decreased TG levels, increased HDL-C content, and modest reductions in TC concentrations [31, 32]. Our docking results suggest that TDPPA possesses a robust binding capacity for PPAR-α, comparable to that of the established positive control. Western blot analysis showed that TDPPA significantly elevated PPAR-α expression relative to the model group. These findings provide both computational and experimental evidence that TDPPA may play a potential role in regulating lipid metabolism through PPAR-α–mediated pathways.

Our results suggest that the lipid-lowering and hepatoprotective effects of TDPPA are mediated through both synergistic and partially independent pathways. The lipid-lowering action is largely driven by PPAR-α upregulation, which mitigates hepatic lipid deposition, alleviates oxidative stress and inflammation, and thereby supports liver function and enhances lipid metabolism in a self-reinforcing therapeutic loop. Independently, TDPPA exerts hepatoprotective effects through modulation of oxidative stress and inflammatory pathways. These protective actions, partly attributable to H_2_S release, can occur even in the absence of significant lipid profile changes. Conversely, PPAR-α activation can improve lipid parameters independently of hepatic injury status. This dual-pathway mechanism—PPAR-α-mediated improvement of lipid metabolism and H_2_S-mediated cellular protection—distinguishes TDPPA from traditional PPAR-α agonists and may account for its superior efficacy observed in this study. Comparable dual activities have been reported for silymarin, a natural compound with antioxidant, anti-inflammatory, and lipid-lowering properties [33, 34].

Comparative analysis demonstrates that TDPPA (15 mg/kg) exhibits approximately two-fold greater hypolipidemic efficacy than compound 6 (13 mg/kg) in high-fat emulsion-induced HLP mice, despite its marginally higher dosage [17]. TDPPA also shows superior anti-inflammatory and antioxidant capacities, achieving greater efficacy at equivalent low/medium/high dose regimens compared to compound T5, as previously described [18]. Computational structural analyses suggest that variations in drug absorption may be involved in treatment outcomes, where TDPPA has a log*P* value of 2.849, compared with compound 6 (3.588) and compound T5 (4.056) [18]. In comparison, the log*P* value of TDPPA appears to make it more suitable for absorption, thereby permitting greater uptake. However, the specific mechanisms remain unclear and warrant further investigation.

This study has several limitations. First, while TDPPA demonstrated hepatoprotective activity in both acute and subchronic hyperlipidemia mouse models and no hepatotoxicity was detected during the 4-week treatment period, the investigation was confined to a subchronic timeframe. A comprehensive evaluation of its long-term safety remains necessary, including chronic toxicity studies, extended pharmacokinetic profiling, and multi-organ toxicity assessments. Second, although TDPPA effectively lowered TG and TC levels while elevating HDL-C levels in hyperlipidemic mice, and appeared favorable relative to the existing lipid-lowering agent Clofibrate, no direct head-to-head comparison was performed. Future studies should therefore incorporate rigorous comparative analyses of efficacy, safety, and pharmacokinetics against standard therapies to more precisely define its therapeutic potential relative to current treatments. Third, the present study was conducted with a relatively small number of biological replicates, which may limit the statistical power and generalizability of the findings. Future work should aim to increase the number of biological replicates to strengthen the robustness and reproducibility of the results.

## CONCLUSION

In conclusion, TDPPA exhibited promising hypolipidemic efficacy in a dose-dependent manner. TDPPA markedly attenuated lipid droplet accumulation and decreased the serum AST and ALT levels in livers, thereby mitigating hepatic injuries in hyperlipidemic mice. Its synergistic antioxidant and anti-inflammatory properties further enhance the therapeutic profile of TDPPA. The hypolipidemic effects of TDPPA are primarily attributed to both the upregulation of PPAR-α expression and the release of H_2_S. Consequently, TDPPA represents a promising dual-action agent with hypolipidemic and hepatoprotective potential.

## Figures and Tables

**Fig. (1):**
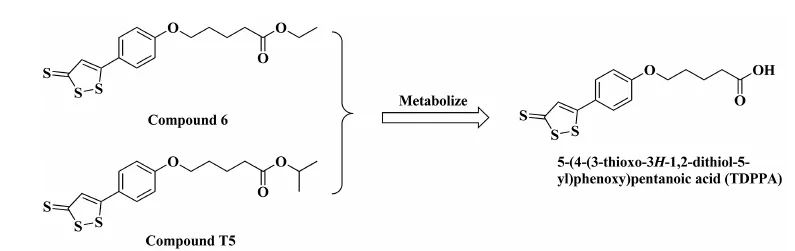
The metabolism of compound 6 and compound T5 results in the production of TDPPA.

**Fig. (2):**
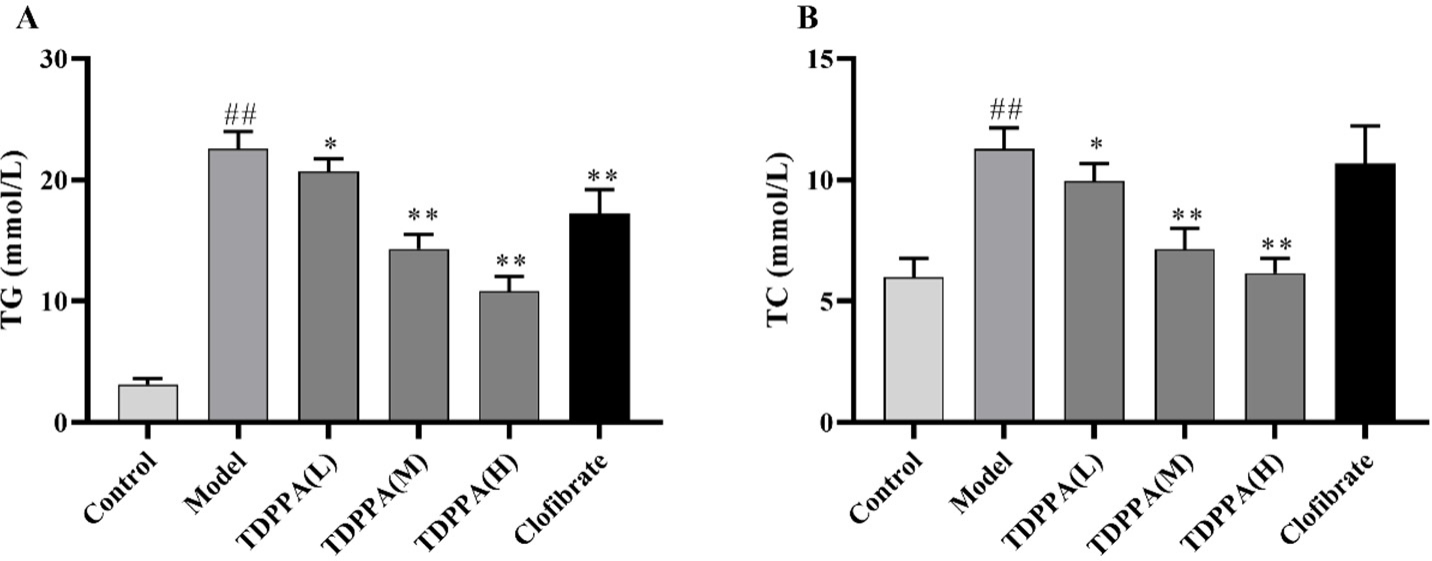
The dose-dependent effects of TDPPA on lipid-lowering activity in Triton WR-1339-induced acute hyperlipidemic mice. (**A**) Serum TG levels; (**B**) Serum TC levels. Data are expressed as mean ± SD (n = 8). ^##^*P* < 0.01 *vs.* control group; *P < 0.05, **P < 0.01 *vs.* model group.

**Fig. (3):**
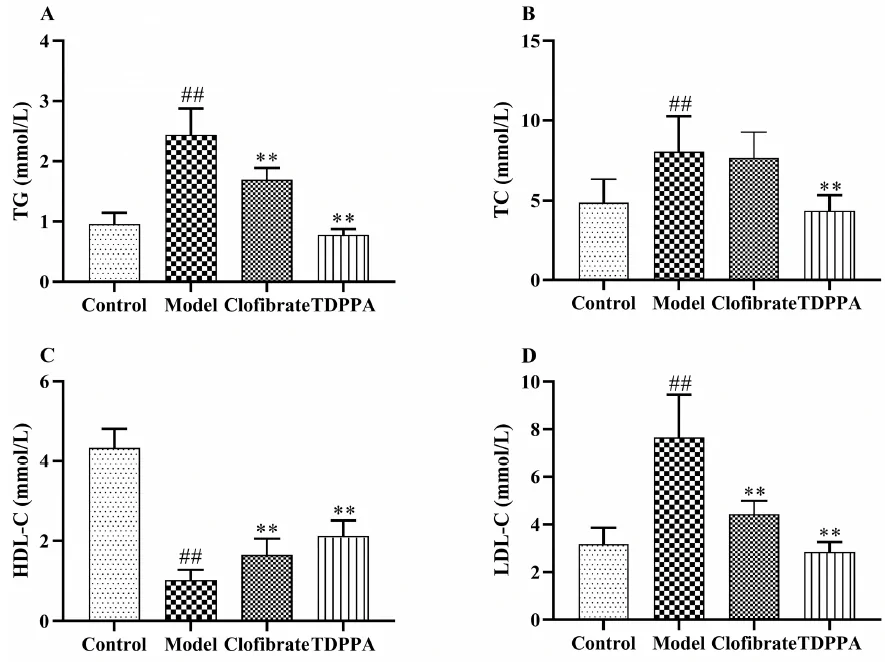
The effects of TDPPA on serum lipid profiles in mice with chronic HLP induced by high-fat emulsion diets. Mice were administered with TDPPA (15 mg/kg) and Clofibrate (65 mg/kg). (**A**) Serum TG levels; (**B**) Serum TC levels; (**C**) Serum HDL-C levels; (**D**) Serum LDL-C levels. Data are expressed as mean ± SD, n = 8. ^##^*P* < 0.01 *vs.* control group; **P < 0.01 *vs.* model group.

**Fig. (4):**
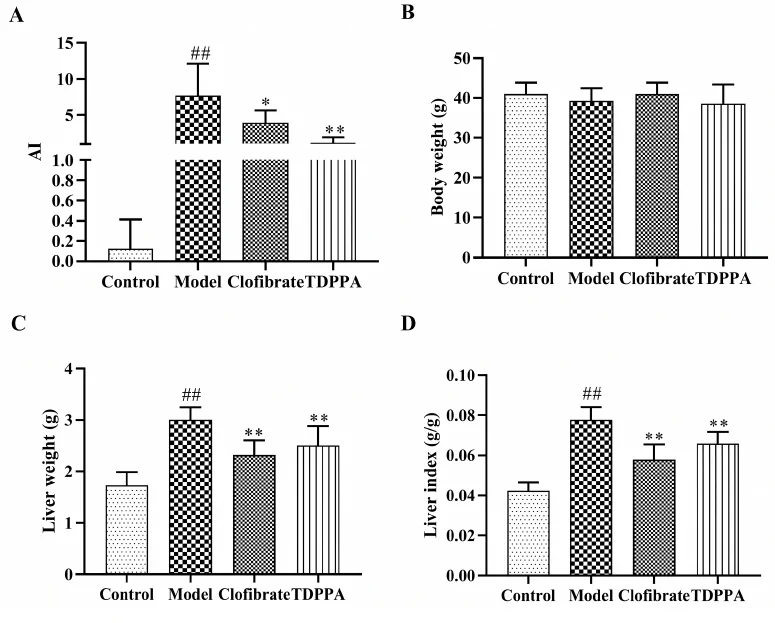
Effects of TDPPA on atherosclerosis index (AI), body weight, liver weight, and liver index (LI) were evaluated in mice with chronic HLP induced by high-fat emulsion diets. Mice were administered with TDPPA (15 mg/kg) and Clofibrate (65 mg/kg). (**A**) Atherosclerosis index (AI); (**B**) Body weight; (**C**) Liver weight; (**D**) Liver index (LI). Data are expressed as mean ± SD, n = 8. ^##^*P* < 0.01 *vs.* control group; *P < 0.05, **P < 0.01 *vs.* model group.

**Fig. (5):**
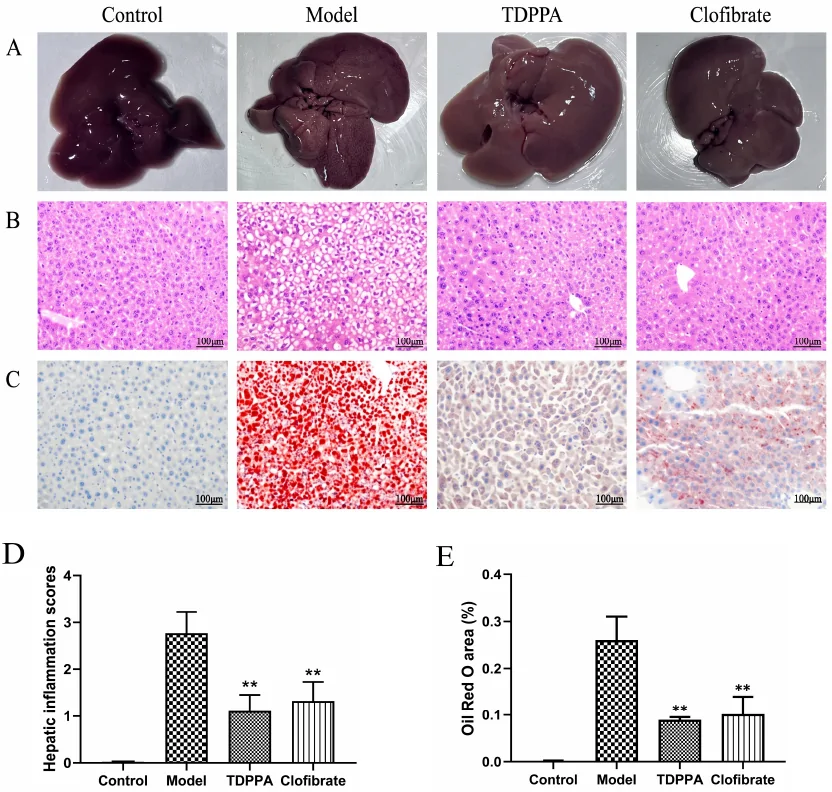
Effects of TDPPA on hepatic morphology and histopathology in mice with chronic HLP induced by high-fat emulsion diets. Mice were administered with TDPPA (15 mg/kg) and Clofibrate (65 mg/kg). (**A**) Macroscopic observation of liver tissues; (**B**) H&E staining (200×); (**C**) Oil Red O staining (200×); (**D**) Histological inflammation scores in each group; (**E**) Quantification of lipid droplets assessed by Oil Red O staining. Data are expressed as mean ± SD, n = 8. **P < 0.01 *vs.* model group.

**Fig. (6):**
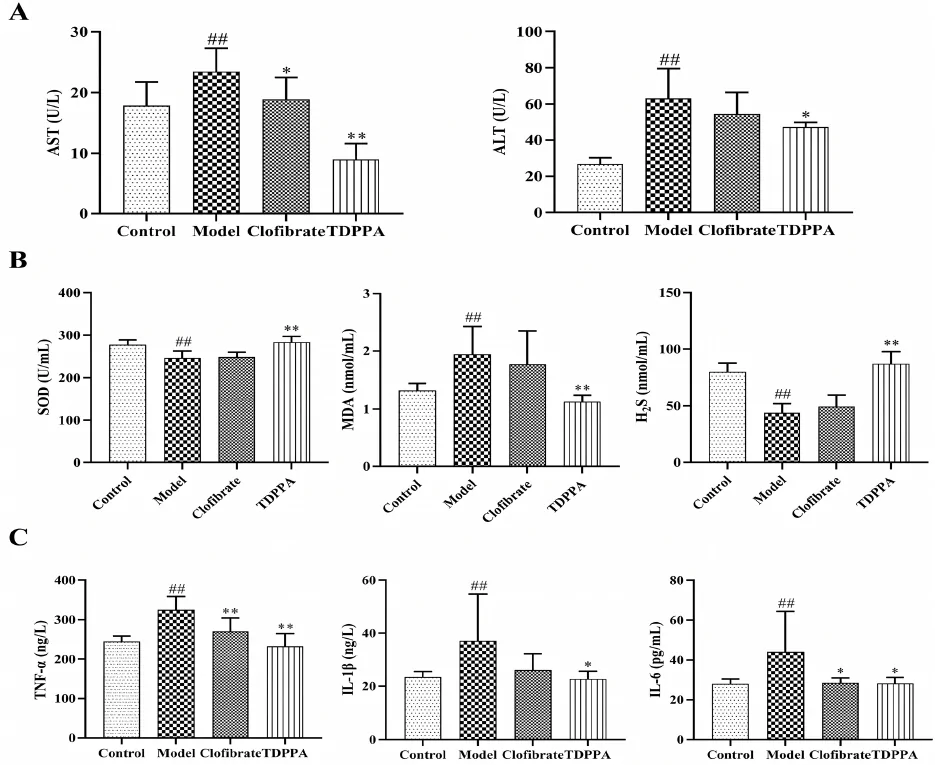
Effects of TDPPA on serum levels of biochemical parameters in mice with chronic HLP induced by high-fat emulsion diets. Mice were administered with TDPPA (15 mg/kg) and Clofibrate (65 mg/kg). (**A**) Serum levels of hepatic injury markers (AST and ALT); (**B**) Serum levels of antioxidant indexes (SOD, MDA, and H_2_S); (**C**) Serum levels of pro-inflammatory cytokines (TNF-α, IL-1β, and IL-6). Data are expressed as mean *±* SD, n = 8. ^##^*P* < 0.01 *vs.* control group; *P < 0.05, **P < 0.01 *vs.* model group.

**Fig. (7):**
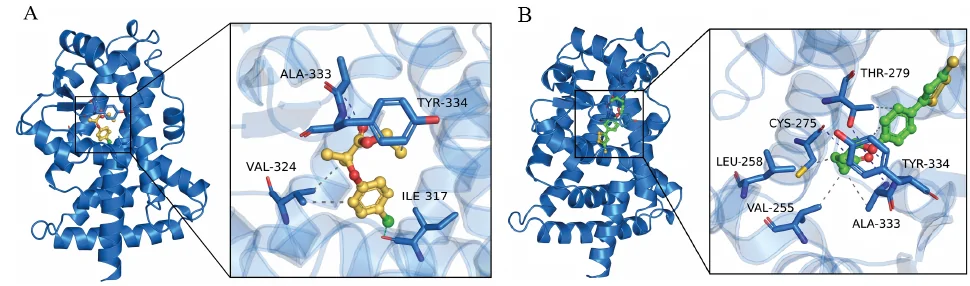
Molecular docking of Clofibrate and TDPPA with the PPAR-α receptor. (**A**) Predicted binding pose of Clofibrate; (**B**) Predicted binding pose of TDPPA.

**Fig. (8):**
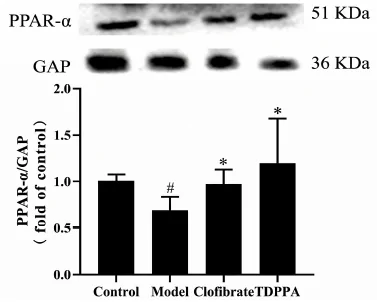
Effect of TDPPA on the expression of PPAR-α in mice with chronic HLP induced by high-fat emulsion diets. Mice were administered with TDPPA (15 mg/kg) and Clofibrate (65 mg/kg). The results are presented as mean ± SD, n = 3. ^#^*P* < 0.05 *vs.* the control group; *P < 0.05 *vs.* the model group.

**Scheme 1:**
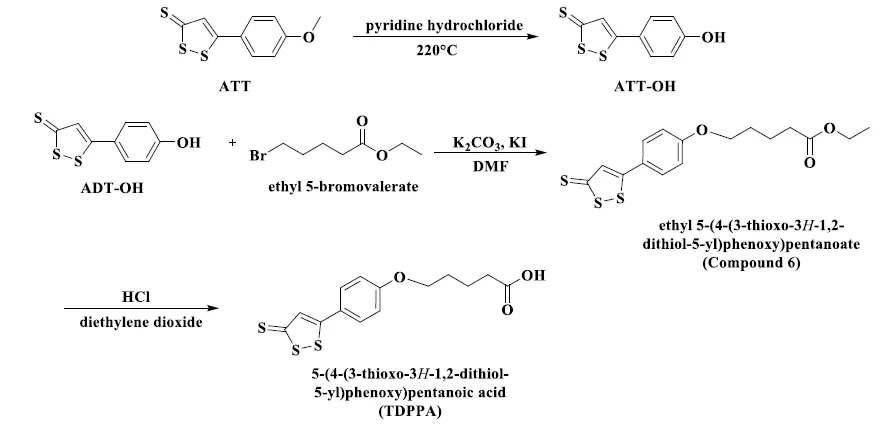
Synthesis of the target compound TDPPA.

## Data Availability

All the data and supporting information are provided within the article.

## LIST OF ABBREVIATIONS

HDL-C: High-Density Lipoprotein Cholesterol
HLP: Hyperlipidemia
HTG: Hypertriglyceridemia
LDL-C: Low-Density Lipoprotein Cholesterol
NAFLD: Non-Alcoholic Fatty Liver Disease
TC: Total Cholesterol
TG: Triglycerides
