# Supplementary material for: Hypolipidemic and Hepatoprotective Effects of 5-(4-(3-thioxo-3H-1,2-dithiol-5-yl)phenoxy)Pentanoic Acid (TDPPA) on Hyperlipidemic Mice
Source: Med Chem. 2025 Oct 8;22(5):E15734064425298. doi: 10.2174/0115734064425298250923050512 (PMC13625030; doi:10.2174/0115734064425298250923050512)
Supplement: Supplementary file 1 [file MC-22-5-E15734064425298_SD1.pdf]

Supplementary Materials

Hypolipidemic and Hepatoprotective Effects of 5-(4-(3-thioxo-3H-1,2-dithiol-5-yl)phenoxy)Pentanoic Acid (TDPPA) on Hyperlipidemic Mice

Ting Li[1], Manjun Liu[1], Qiuzhen Yuan[1], Wenjing Shen[2], Xueyou Chen[3], Lei Zhang[1,*] and Yundong Xie[4,*]

[1]Pharmaceutical Department, Shaanxi Institution of Traditional Chinese Medicine, Shaanxi Traditional Chinese Medicine Hospital, Xi'an, China; [2]Department of Pharmacy, Pengzhou City Fourth People's Hospital, Pengzhou Mental Health Center, Pengzhou, China; [3]Department of Pharmacy, Zigong Hospital of Traditional Chinese Medicine, Zigong, China; [4]School of Pharmacy, Shaanxi University of Chinese Medicine, Xianyang, China

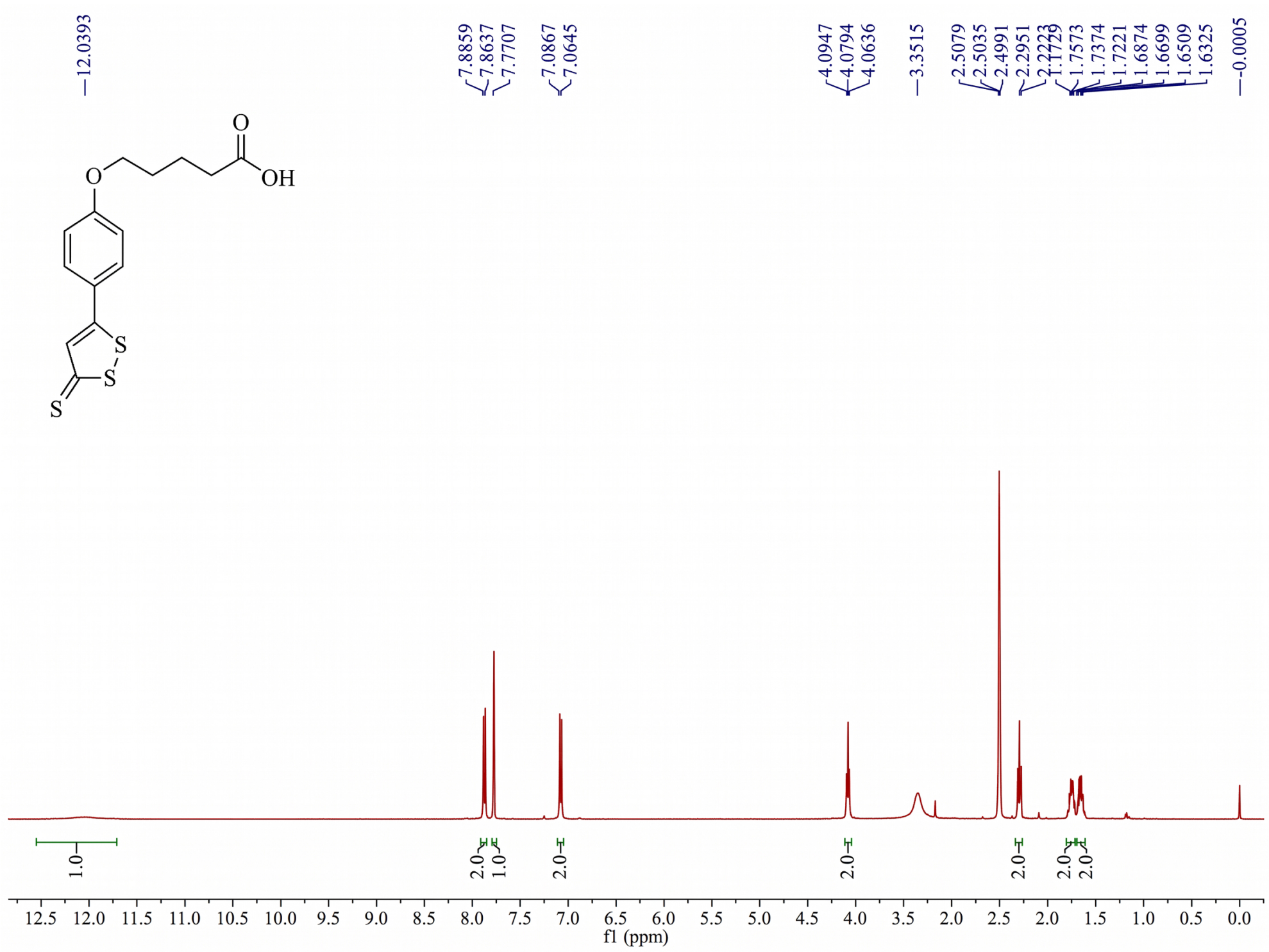

^{1}H-NMR spectrum of the compound TDPPA

 © 2026 Bentham Science Publishers

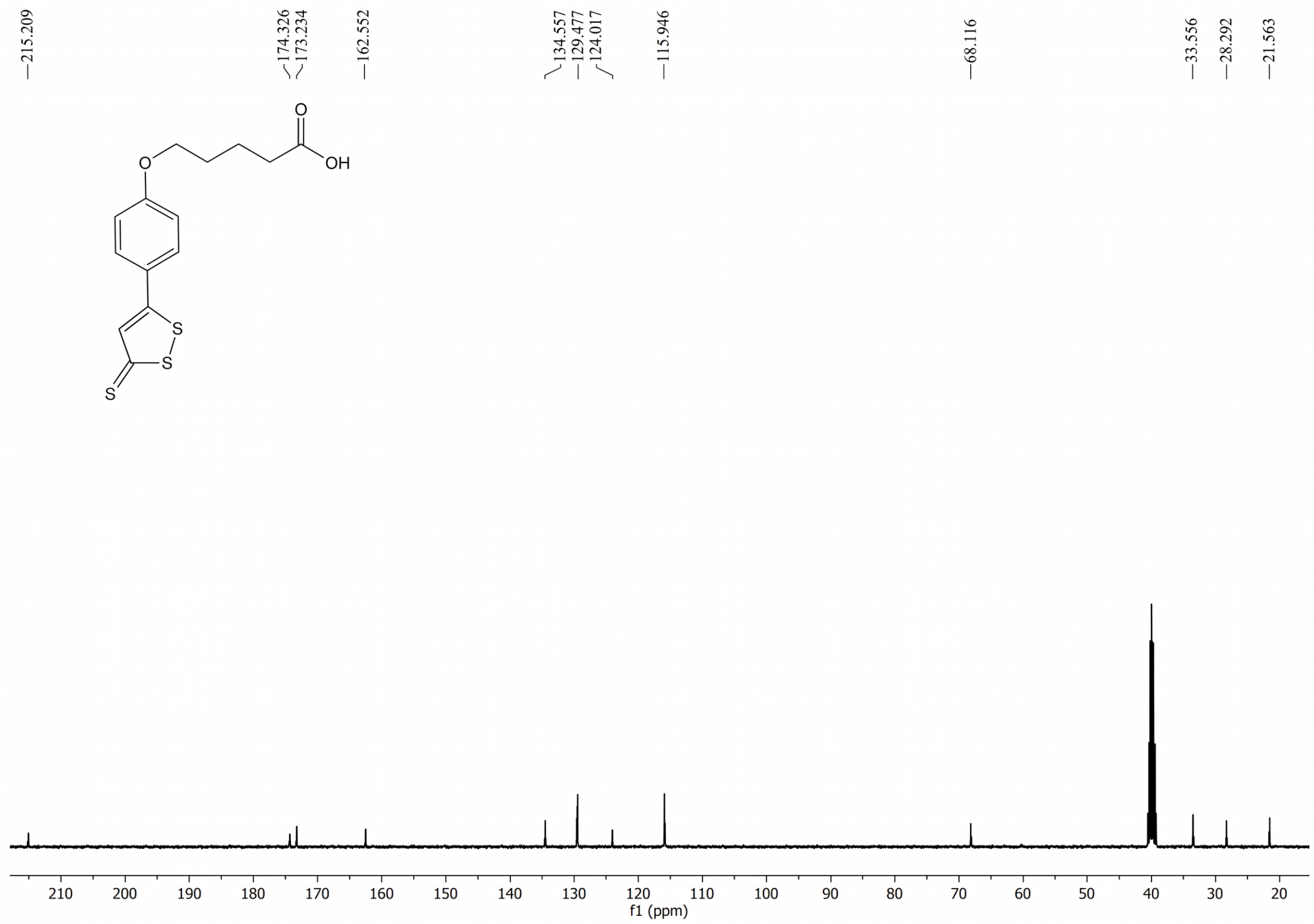

The ^{13}C-NMR spectrum of the compound TDPPA

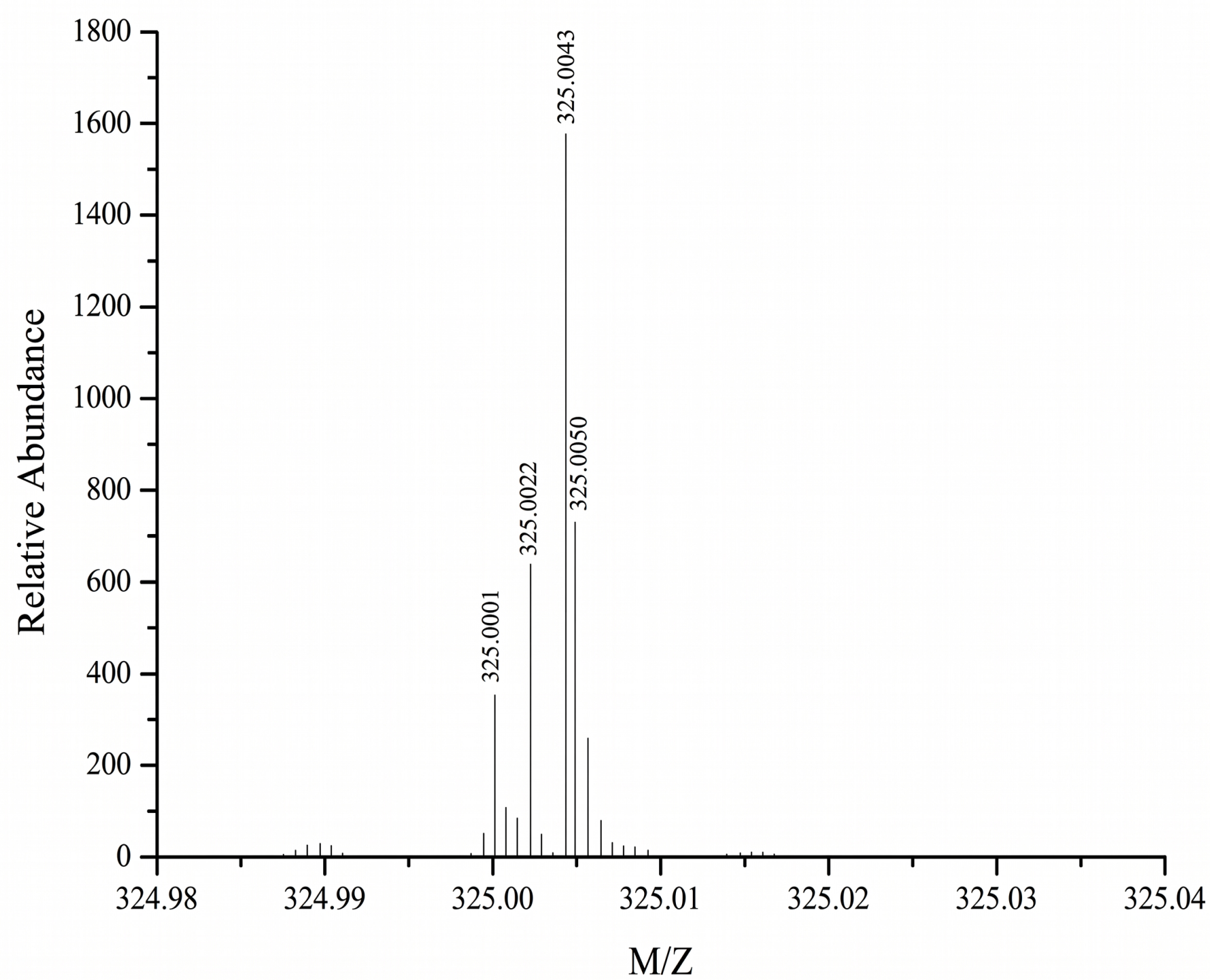

The HR-MS spectrum of compound TDPPA.
